# Breast Metastasis of Lung Cancer After Computed Tomography-Guided Core Needle Biopsy: A Case Report

**DOI:** 10.3389/fsurg.2022.890492

**Published:** 2022-04-26

**Authors:** Rong Zhao, Jun Xing, Jinnan Gao

**Affiliations:** Department of Breast Surgery, Shanxi Bethune Hospital, Shanxi Academy of Medical Sciences, Tongji Shanxi Hospital, Third Hospital of Shanxi Medical University, Taiyuan, China

**Keywords:** breast, case report, lung neoplasms, neoplasm seeding, surgery

## Abstract

**Background:**

Needle tract metastasis is a rare complication following percutaneous procedures for malignancy.

**Case Summary:**

This report describes a 49-year-old female with a lump on her right breast. Mass core needle biopsy showed the specimen was an invasive carcinoma, and mastectomy with sentinel lymph node biopsy was performed. What is special about this case was that the patient reported a history of lung cancer and the position of the breast mass was the puncture site of computed tomography-guided core needle biopsy for lung cancer. Immunostaining of paraffin specimen findings indicated the breast mass as a result of lung carcinoma metastasis. The patient's medical history indicated that the malignant tumor in the breast was a core needle tract pulmonary metastasis. The patient underwent the examination and received therapy based on the lung cancer metastasis principle. At 9 months from breast surgery, the patient is alive, in good condition, and with stability of the disease.

**Conclusions:**

This patient was misdiagnosed. Careful medical history review and multidisciplinary team discussions are important, especially for patients with a history of cancer or invasive operation.

## Introduction

Breast cancer is the most common malignant tumor in women, but metastasis to the breast from extramammary malignancies is rare (about 0.4–2%) (1). Previous reports (1, 2) showed breast cancer is most often secondary to lung cancer, cutaneous melanomas, malignant lymphomas, and primary tumors of the esophagus, retina, pancreas, thyroid, and skin. Lymphatic metastasis and hematogenous metastasis were the reported common types of cancer metastasizing to the breast. To our knowledge, this is the first case report describing a lung core needle tract metastatic to the breast.

## Case Report

A 49-year-old patient was admitted to the breast department after presenting with a lump on her right breast in June 2021. Palpation of the mass for 8 months showed a gradual increase in diameter to >2 cm. Physical examination showed the presence of a firm, hard, and immovable mass at the 12-o'clock position of the breast 7 cm from the nipple. Mammogram, ultrasound, and magnetic resonance imaging (MRI) were performed to further explore the mass. The results showed a high possibility of malignancy and the mass was classified as Breast Imaging Reporting and Data System 5 (BI-RADS: 5).

**Figure 1:**
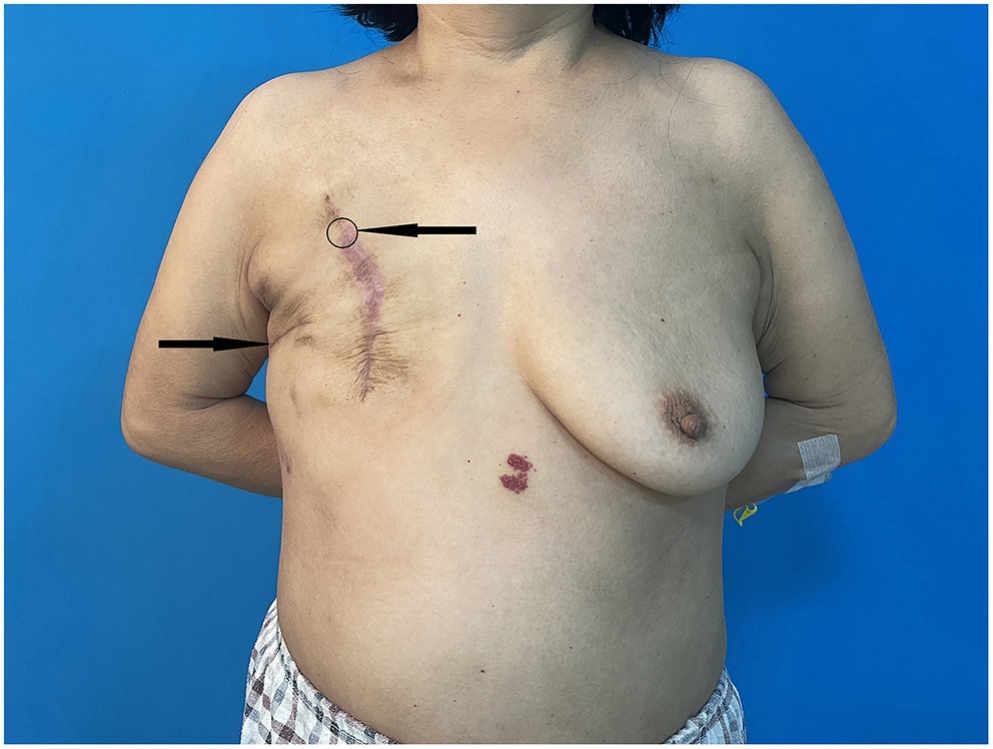
Post-operative photo indicating the patient's breast mass position (arrow and circle) as well as the previous incision for lung cancer (arrow).

Mass core needle biopsy under ultrasound guidance was performed and routine pathology showed the specimen was an invasive carcinoma. The patient rejected breast-conserving surgery, therefore, mastectomy with SLNB (sentinel lymph node biopsy) was performed (Figure 1).

**Figure 2:**
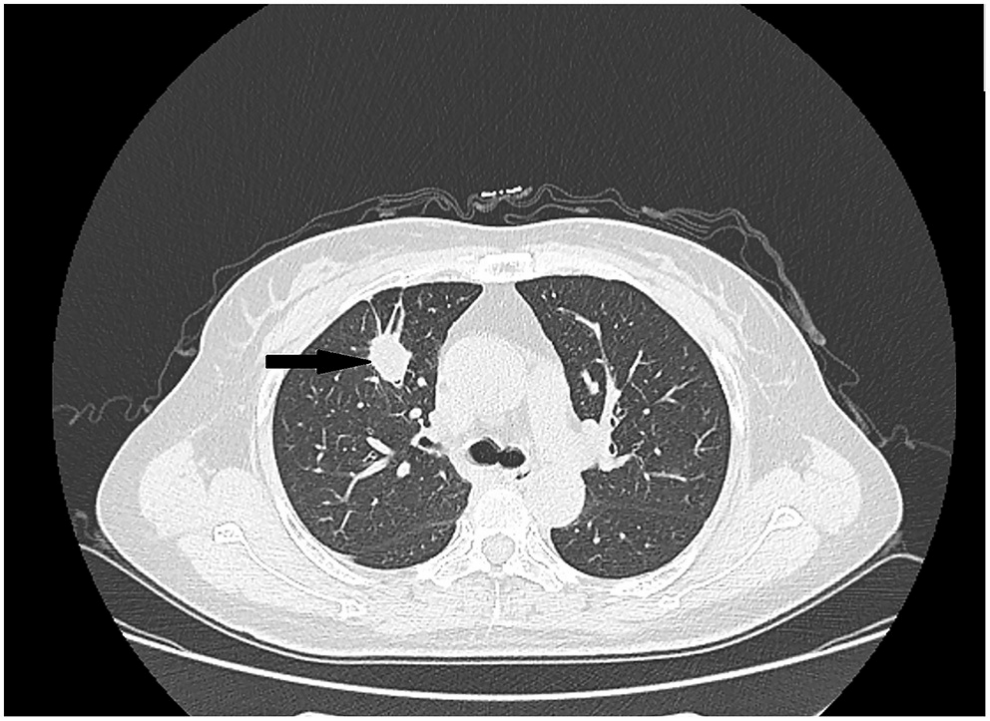
Computed tomography scan showing the primary lung tumor (arrow).

**Figure 3:**
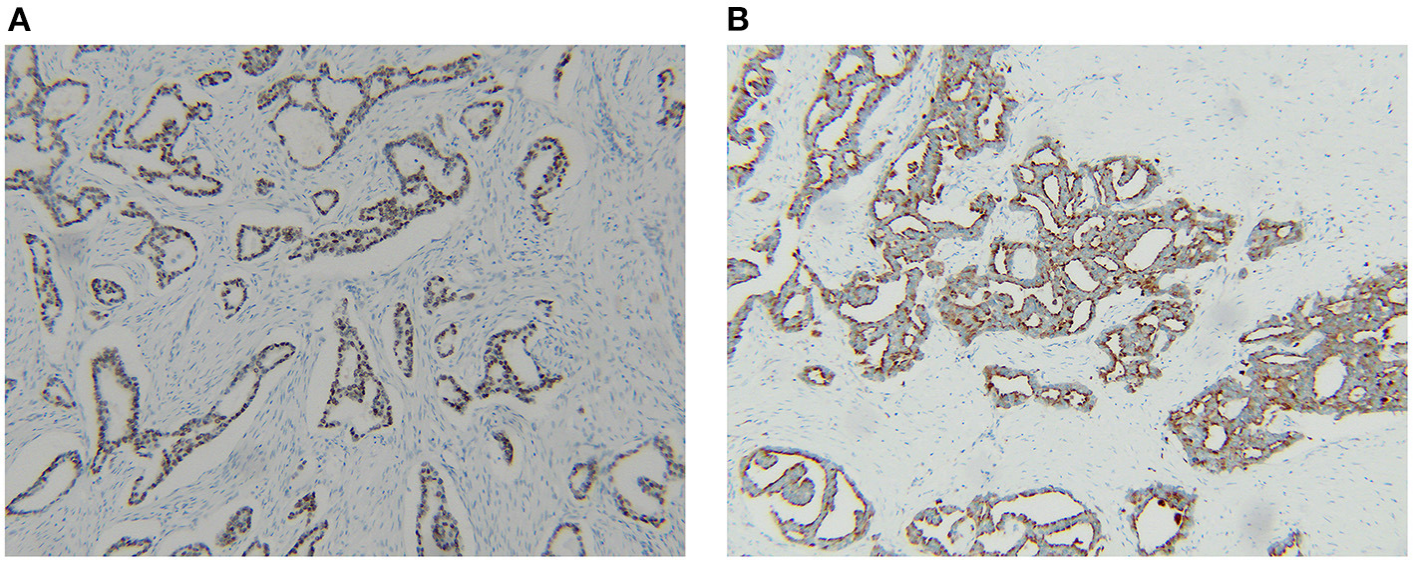
Expression of TTF-1 **(A)** and napsin A **(B)** indicating the breast lump was a metastasis from a lung primary cancer.

Notably, the patient reported a history of lung cancer diagnosed in January 2019. Computed tomography (CT) revealed a lump in the superior lobe of the right lung (Figure 2) and adenocarcinoma of the lung was diagnosed through the 18-G conventional core needle biopsy (Biopty, Bard, Covington, GA, USA). Physical examination, laboratory findings and other exams showed no positive signs. So, complete resection was performed. The pathological diagnosis of the specimens was a 2 × 1.5 × 1 cm lung adenocarcinoma, without the involvement of the resection margins, chest wall muscles, and fascia, and with no perineural invasion and lymph node metastasis. The results of immunohistochemistry (IHC) staining were positive for Napsin A, TTF-1 (Figure 3), and CK7 and negative for p40, Syn. Epidermal growth factor receptor (EGFR) was locally weakly positive and Ki67 was 30%. Chemotherapy and radiation were not conducted because of the early stage of NSCLC (non-small-cell lung cancer) IA2 without the involvement of the resection margins. The patient was followed up regularly every 6 months and a chest CT performed in September 2020 showed no abnormality. One month later (October 2020), she detected a breast mass the size of a peanut grain but did not seek medical attention. The breast mass was evident in CT when she went for routine follow-up in June 2021 and the thoracic surgeon referred her to the breast department.

After breast surgery, paraffin pathological report showed the breast resection margins were clear. Results from immunohistochemical staining for estrogen and progesterone receptors were negative, CerbB-2 showed 2+ positivity and Fluorescence In Situ Hybridization (FISH) was not amplified. Thyroid transcription factor (TTF-1) and napsin A were performed owing to the lung cancer history and positive results were obtained, indicating lung cancer metastasis. These findings indicated that the breast mass was a result of metastasis of the lung adenocarcinoma to the breast.

After being informed, the patient reported that the position of the breast mass was the puncture site of computed tomography-guided core needle biopsy for lung cancer. Previous CT images were reviewed and the position corresponded to the puncture site. The patient's report and pathological results indicated that the malignant tumor in the breast was a needle tract metastasis of pulmonary adenocarcinoma. The patient underwent the examination and received therapy based on the lung cancer metastasis principle. Currently, the patient has undergone chemotherapy and radiation therapy. At 9 months from breast surgery, the patient is alive, in good condition, and with stability of the disease.

## Discussion

Pre-operative needle biopsy is a useful approach for the diagnosis of lung cancer owing to its high accuracy and few complication rates (3, 4). Needle track pulmonary metastases are rare (5). There were a few case reports (5–7) describing a lung needle-tract metastatic to the chest. A previous report of implantation metastasis to the mammary gland was the complication of fine-needle aspiration biopsy (FNAB) (8), so this is the first report of a lung core needle-track metastatic to the breast. Compared with FNAB, core needle biopsy (CNB) has greater diagnostic accuracy, however, FNAB under CT guidance is subject to a lower rate of complications (9). It is important to note that the indicated complications mainly include pneumothorax and parenchymal hemorrhage, not including implantation metastasis.

It is challenging to distinguish primary breast cancer from metastatic adenocarcinoma through the clinical presentation, radiological findings, and routine pathology (1). However, immunohistochemistry is accurate in the diagnosis of cancer types. TTF-1 and napsin A have high positive predictive value (PPV) and diagnostic accuracy for adenocarcinoma of the lung (10). In the current case, the pathologist did not identify the source of the breast puncture specimen for routine hematoxylin and eosin-stained section showed adenocarcinoma with no specific distinguishing characteristics, thus the patient received treatment for primary breast cancer resulting in a large surgical scope. Clinical history is often sufficient for the pathologist to diagnose a lump as primary or metastatic, however, clinicians didn't emphasize the specificities of the case.

This patient's lung cancer history was provided to the pathologist after breast surgery. The lung cancer history of the patient formed a basis for analysis of TTF-1 as well as napsin A biomarkers in paraffinized specimens. Breast lesions were diagnosed on time as metastasis, so oligorecurrence occurred in this patient who had undergone curative-intent surgery to lung cancer. Systemic therapy is the standard palliative treatment for patients with NSCLC after relapsing to distant sites. The benefit of treating patients with systemic therapy for NSCLC with oligometastases amenable to resection is not well understood, but in extrapolating from the benefit of systemic therapy for locoregionally advanced and metastatic NSCLC, systemic therapy to those with oligometastatic NSCLC is generally offered (11). Surgery (12) and/or radiotherapy (13) may also be combined with systemic therapy. Decisions between radiotherapy and surgery, or potentially how to combine them are usually made in a multidisciplinary tumor board. Based on the above principle, the patient received the appropriate subsequent evaluation, chemotherapy, and radiotherapy. Distinguishing between metastatic disease to the breast and primary lung cancer is important, as it is about the treatment and the outcome.

This patient's lesion presented as a triple-negative adenocarcinoma. In a previous study (12), the lesion metastatic to the breast was also triple-negative. This raises the question of whether breast cancer presented as a triple-negative characteristic might be metastatic adenocarcinoma (not including metastasis from contralateral breast carcinoma). Another concern is the puncture point. Since the case of the lung needle-track metastatic to the breast is rare and it is not clear what the clinical risk factors are associated with cancer seeding after core needle biopsy, further studies should explore is it more prone to metastasize along the needle tract when involving the mammary gland for lung cancer diagnosis.

Thoracic or breast surgeons should carefully review the entire previous diagnosis and treatment process with such cases, thus avoiding errors in diagnosis and treatment. In addition, medical history is significant for the pathologist, detailed recordings can influence their ability to make correct judgments, which would, in turn, guide clinicians to take the correct decision about treatment. In summary, careful medical history review and multidisciplinary team (MDT) discussions are essential, especially for patients with a history of cancer and invasive operations.

## Data Availability Statement

The original contributions presented in the study are included in the article/supplementary material, further inquiries can be directed to the corresponding author.

## Ethics Statement

The studies involving human participants were reviewed and approved by Ethics Committee of Shanxi Bethune Hospital. Written informed consent was obtained from the individual for the publication of any potentially identifiable images or data included in this article.

## Author Contributions

RZ and JX: writing—original draft. JG: writing—review and editing. All authors contributed to the article and approved the submitted version.

## Funding

This article was sponsored by the 136 Medical Project by Shanxi Provincial Government.

## Conflict of Interest

The authors declare that the research was conducted in the absence of any commercial or financial relationships that could be construed as a potential conflict of interest.

## Publisher's Note

All claims expressed in this article are solely those of the authors and do not necessarily represent those of their affiliated organizations, or those of the publisher, the editors and the reviewers. Any product that may be evaluated in this article, or claim that may be made by its manufacturer, is not guaranteed or endorsed by the publisher.
